# Properties and Potential Antiproliferative Activity of Thrombin-Binding Aptamer (TBA) Derivatives with One or Two Additional G-Tetrads

**DOI:** 10.3390/ijms232314921

**Published:** 2022-11-29

**Authors:** Daniela Benigno, Antonella Virgilio, Ivana Bello, Sara La Manna, Valentina Vellecco, Mariarosaria Bucci, Daniela Marasco, Elisabetta Panza, Veronica Esposito, Aldo Galeone

**Affiliations:** Department of Pharmacy, University of Naples Federico II, I-80131 Napoli, Italy

**Keywords:** G-quadruplex, thrombin-binding aptamer, abasic site mimic, antiproliferative G-rich oligonucleotides, nucleolin

## Abstract

In this paper, we study the biological properties of two TBA analogs containing one and two extra G-tetrads, namely TBAG3 and TBAG4, respectively, and two further derivatives in which one of the small loops at the bottom (TBAG41S) or the large loop at the top (TBAG4GS) of the TBAG4 structure has been completely modified by replacing all loop residues with abasic site mimics. The therapeutical development of the TBA was hindered by its low thermodynamic and nuclease stability, while its potential as an anticancer/antiproliferative molecule is also affected by the anticoagulant activity, being a side effect in this case. In order to obtain suitable TBA analogs and to explore the involvement of specific aptamer regions in biological activity, the antiproliferative capability against DU 145 and MDAMB 231 cancer cell lines (MTT), the anticoagulant properties (PT), the biological degradability (nuclease stability assay) and nucleolin (NCL) binding ability (SPR) of the above described TBA derivatives have been tested. Interestingly, none of the TBA analogs exhibits an anticoagulant activity, while all of them show antiproliferative properties to the same extent. Furthermore, TBAG4 displays extraordinary nuclease stability and promising antiproliferative properties against breast cancer cells binding NCL efficiently. These results expand the range of G4-structures targeting NCL and the possibility of developing novel anticancer and antiviral drugs.

## 1. Introduction

Aptamers are DNA- or RNA-based ligands, sometimes discovered by chance but more often identified through several combinatorial techniques collectively called SELEX (Systematic Evolution of Ligands by EXponential enrichment) [1]. Depending on their sequence, aptamers can generally adopt specific three-dimensional structures that allow them to interact with a given target with high affinity and specificity. A not negligible number of aptamers folds in G-quadruplex structures (G4-aptamers), being these unusual DNA/RNA conformations often endowed with remarkable thermal stabilities [2,3]. Many G4-aptamers are known to possess promising anticoagulant, antiviral or antiproliferative properties [4]. Among these, the thrombin-binding aptamer (TBA) is particularly noteworthy since it is endowed with both anticoagulant [5] and antiproliferative activities [6].

Unfortunately, the therapeutical development of the unmodified TBA as an anticoagulant agent halted, mainly due to some unfavorable aspects, such as the limited stability both from a thermodynamic point of view and in biological environments [7]. On the other hand, the potential exploitation of TBA as an anticancer/antiproliferative molecule, beside is affected by the same drawbacks described before, must also consider the anticoagulant activity, which, in this case, should be seen as a side effect. For these reasons, a significant part of the research focused on the antiproliferative activity of TBA has been addressed with chemical modifications aimed at increasing thermal and biological resistance and weakening the anticoagulant activity without affecting the antiproliferative one. For example, the replacement of thymidine in position 13 (located in one of the small loops) with a dibenzyl linker provides the modified TBA with significant antiproliferative activity against HeLa cervical carcinoma cells but lacking in anticoagulant activity [8]. Similarly, the replacement of thymidines in positions 4 and 13 with 5-hydroxymethyl-deoxyuridine residues results in TBA analogs endowed with antiproliferative activity against lung cancer (Calu-6) and colorectal cancer cells (HCT 116^p53−/−^) but having a negligible residual anticoagulant activity [9]. Encouraging results have also been obtained by heavier modifications involving the sugar moiety. For example, the incorporation of 4-thiouridine RNA and 4-thiouridine unlocked nucleic acid (UNA) residues in definite positions dramatically decreases the anticoagulant activity but allows the modified TBA to acquire a noteworthy antiproliferative activity against HeLa cervical carcinoma cells [10]. Interestingly, more radical backbone alterations (for example, the introduction of inversion of polarity sites) can afford modified TBA endowed with antiproliferative activity against Calu-6 and HCT 116^p53−/−^ cells but with no anticoagulant properties [11,12]. Recently, TBA analogs have been described as formed by four pairs of enantiomeric heterochiral compounds, each containing only an L-residue in a sequence mostly composed of D-residues or vice versa [13]. The TBA derivatives of the L-series showed remarkable cytotoxic activities against colon and lung cancer cell lines but no anticoagulant activity. Importantly, they were also considerably resistant in biological environments.

According to literature data, the antiproliferative/cytotoxic activity of TBA has been suggested to depend on at least two potential biological pathways, namely, the cytotoxicity of guanine-based degradation products of guanine-rich oligonucleotides [14,15] and the processes mediated by thrombin and associated with cancer [16,17]. However, some investigations concerning antiproliferative TBA analogs undegradable in biological environment and/or characterized by no anticoagulant activity [11,13] suggest that alternative biological pathways could contribute to the antiproliferative activity by the involvement of one or more still unidentified targets, similarly to what has been already observed for a promising antiproliferative G4-aptamer, namely AS1411, which has been proven to interact with nucleolin (NCL) [18].

The above observations clearly suggest that a suitable TBA analog useful to investigate more specific molecular pathways involved in antiproliferative activity should be endowed with the following features: (1) high thermal stability; (2) outstanding resistance in biological environments; and (3) no ability to interact with the thrombin and then, lacking in anticoagulant activity. Since most of the thermal stability of a G-quadruplex structure depends on the number of stacked G-tetrads, an interesting approach to obtain more stable TBA derivatives is increasing the G-residues in the G-tracts involved in the formation of the G-quadruplex stem.

**Figure 1:**
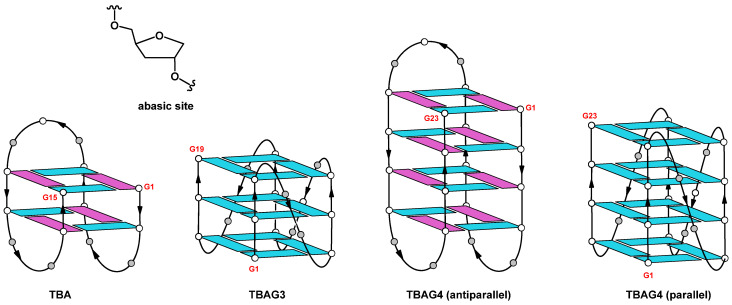
Schematic representation of the G4 structures adopted by the studied sequences and chemical structure of the abasic site mimic (S). Guanosines adopting *anti* and *syn* glycosidic conformations are in light blue and purple, respectively. Gray circles represent loop residues or abasic sites in the TBAG4 parallel structure, according to the sequences (Table 1).

In this paper, we have investigated the antiproliferative activities against DU 145 and MDAMB 231 cancer cell lines, the anticoagulant properties, and the biological degradability of two TBA analogs, namely TBAG3 and TBAG4 (containing one and two extra G-tetrads more, respectively) and those of two further derivatives in which one of the small loops at the bottom (TBAG41S) or the large loop at the top (TBAG4GS) of the TBAG4 structure has been completely modified by replacing all loop residues with abasic site mimics (Table 1, Figure 1), with the aim to obtain insight into the role of these G-quadruplex regions in the biological activity. Furthermore, the affinity of TBA and its analogs to NCL was investigated by surface plasmon resonance (SPR) (Table 1).

## 2. Results

### 2.1. Structural Insights of the Investigated Sequences

**Figure 2:**
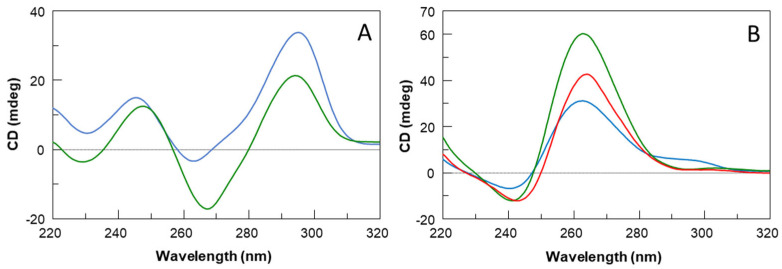
CD spectra at 20 °C in potassium phosphate buffer (10 mM KH_2_PO_4_/K_2_HPO_4_, 70 mM KCl, pH 7.0) at 50 µM ODN strand concentration of (**A**) TBA (green) and TBAG4 (blue); (**B**) TBAG4GS (green), TBAG41S (blue) and TBAG3 (red).

It is well known that TBA folds in a monomolecular antiparallel “chair-like” G-quadruplex structure characterized by two stacked *syn-anti-syn-anti* G-tetrads and three lateral loops (Figure 1) [19]. On the other hand, based on CD data previously reported, the TBA analog containing an extra G-residue in each G-run of the sequence, namely TBAG3 (Table 1), has been suggested to adopt a major parallel G-quadruplex structure in potassium buffer with three all-*anti* G-tetrads and three propeller loops (Figure 1) [20,21]. Surprisingly, earlier NMR and CD studies have shown that the insertion of further guanosine in each G-run of the sequence (TBAG4) results again in the formation of an antiparallel G-quadruplex structure with four stacked *syn-anti-syn-anti* G-tetrads and three lateral loops very similar to those observed in the original TBA G-quadruplex structure (Figure 1) [22]. In order to explore the importance of the G-quadruplex loop regions in the biological activity (see below), two other sequences were investigated, namely TBAG41S and TBAG4GS, in which all residues in one small loop or in the large loop, respectively, were replaced with abasic site mimics (Table 1). As in the case of the previous sequences, the CD spectrum represents a straightforward tool to ascertain the occurrence of G-quadruplex structures and obtain information about their relative strand orientation. CD spectra of the investigated sequences are shown in Figure 2A,B. Profiles of TBAG41S and TBAG4GS are both characterized by a negative band and a positive one around 243 and 265 nm, respectively, which are indicative of parallel G-quadruplex structures where all guanosines adopt *anti*-glycosidic conformations. However, the CD profile of TBAG41S also shows a shoulder at 295 nm, suggestive of the presence of minor amounts of antiparallel species.

CD technique was employed to evaluate the structural thermal stability (Table 1). Figure S1 shows the CD heating profiles of the G-quadruplex structures under investigation. The sigmoidal profiles of CD signals *versus* temperature for TBAG3 and TBAG4GS allowed the evaluation of their melting temperatures (T_m_, 77 and 85 °C, respectively). On the contrary, the CD heating curve of TBAG41S shows a sub-optimal profile, which only allowed us to roughly estimate the melting temperature higher than 70 °C. Interestingly, the heating curve of TBAG4 shows that the melting process just starts around 90 °C, thus clearly indicating outstanding thermal stability for this structure.

The G-quadruplex structures were further analyzed by polyacrylamide gel electrophoresis (PAGE) (Figure S2). The parent G-quadruplex TBA was used as a reference. TBAG3 shows a unique band with lower motility than TBA. These data are consistent with those suggested by the CD profile indicating the formation of a parallel monomolecular G-quadruplex structure with propeller loops contributing to reducing the mobility. TBAG4 shows the main band, although the presence of higher-order structures. However, unexpectedly, the mobility of the main band is comparable to that of TBA, notwithstanding the larger size of the G-quadruplex structure suggested by CD and NMR data. This result could be explained by the high compactness of both structures due to the absence of propeller loops. The electrophoretic behavior of the other two G-quadruplex structures is quite different from that of TBAG3 and TBAG4. TBAG4GS shows both an evident smearing near the well, being suggestive of higher-order structures, and a band slower than that of TBAG3, probably due to a parallel monomolecular structure, as suggested by the CD profile (Figure 2B). On the other hand, in the case of TBAG41S, the smearing is more marked compared to that of TBAG4GS, while there is only a barely visible fast-migrating band. In this case, CD and PAGE data agree in suggesting the presence of the main amount of higher-order structures and only a negligible quantity of monomolecular G-quadruplex structures.

### 2.2. Nuclease Stability Assay

**Figure 3:**
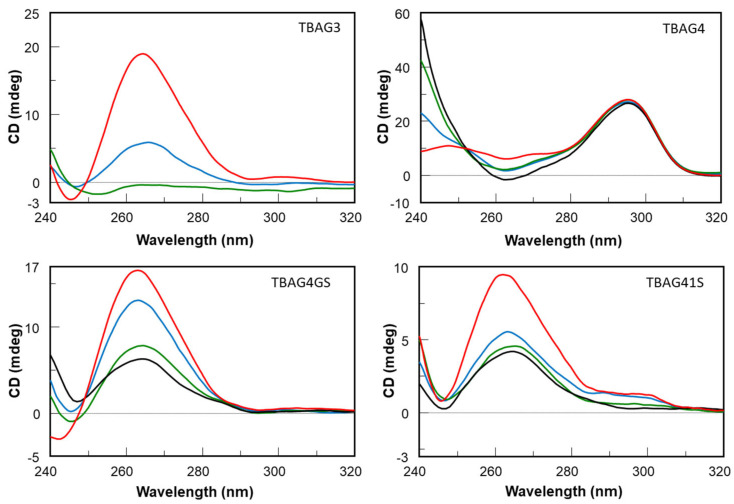
CD spectra of modified ODNs in 10% FBS diluted with Dulbecco’s Modified Eagle’s Medium (DMEM), registered at 0 (red), 24 h (blue), 48 h (green), and 72 h (black), at 37 °C.

The stability of nucleic acid aptamers is one of the most important elements for their potential use in therapeutic applications. An aptamer with suitable stability under physiological conditions can be directly used for biomedical applications without further optimization, thus decreasing financial costs considerably. Therefore, to test the resistance in biological environments, all the investigated ODNs were undergone to a degradation assay in fetal bovine serum (FBS) and were analyzed at different times by CD (Figure 3) in comparison with the unmodified aptamer TBA (Figure S3) [13]. To check the persistence over time of the CD signal due to undegraded G-quadruplex structures in solutions, CD spectra of all ODNs have been registered in 240–320 nm region at 0, 24, 48, and 72 h at 37 °C in 10% FBS, that contains greater than 256 U/L equivalent of DNase I activity, as previously reported by Hahn J. and coauthors [23]. After subtraction of the background scan (10% FBS in DMEM), most of the analyzed aptamers showed CD signals decreasing in a time-dependent manner. CD spectra of the parent TBA have been acquired at different time points since, in the same experimental conditions, the results almost completely degraded already at 30 min (Figure S3). Most marked effects are evident on TBAG3, which is completely degraded after 48 h, whereas TBAG41S and TBAG4GS exhibit a similar partial resistance to nucleases since 35–40% of G-quadruplex species are still present at 72 h in both cases. The most interesting results were obtained for TBAG4, which revealed a noteworthy improvement in nuclease resistance in comparison to the other analogs, since the CD band intensity of TBAG4 at 295 nm, typical of TBA-like antiparallel G-quadruplex, remains unchanged over time. These data indicate that, under the experimental conditions reported, this TBA analog is still stable after 72 h, thus providing the highest resistance to nucleases ever observed for natural G-quadruplex-forming oligonucleotides, to the best of our knowledge.

### 2.3. Anticoagulant Activity

To evaluate the anticoagulant activity of TBA derivatives, the compounds were subjected to a prothrombin time (PT) assay. Unlike the reference TBA compound, the results clearly revealed that the addition of one (TBAG3) or two (TBAG4) extra G-tetrads completely abolished the anticoagulant activity, bringing it back to the values observed in the vehicle (Figure S4). In detail, at the concentration of 20 µM, the original TBA showed a PT value of 60.5 ± 3.8 s; meanwhile, TBAG3 and TBAG4 showed a PT value of 16.3 ± 1.3 s and 15.2 ± 1.9 s, respectively.

### 2.4. Antiproliferative Activity

**Figure 4:**
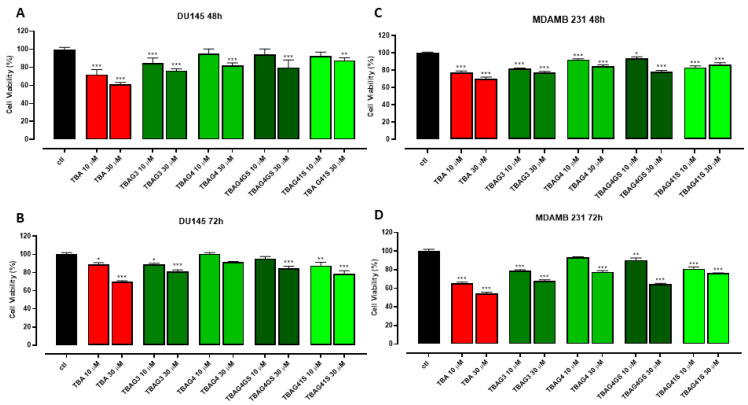
Effect of TBA and its analogs on DU 145 (**A**,**B**) and MDAMB 231 (**C**,**D**) cell proliferation. Cell proliferation was measured using the MTT assay and evaluated at 48 and 72 h. Each experiment was run in quadruplicate. * *p* < 0.05; ** *p* < 0.01; *** *p* < 0.001 vs. CTL.

To test the potential anticancer action of TBA analogs, the proliferation of both human prostate cancer (DU 145) and breast cancer (MDAMB 231) cells was evaluated upon exposition to four distinct TBA analogs (TBAG3, TBAG4, TBAG4GS, and TBAG41S) in comparison with the parent TBA. In DU 145 cells at 10 µM, only TBAG3 and TBAG41S exhibited an antiproliferative activity comparable to that of TBA after 72 h with a reduction in cell viability of 10%. Conversely, at 30 µM, both after 48 and 72 h, all four TBA analogs showed an enhancement of their anti-proliferative activity, although lower than that of TBA (Figure 4A,B). Notably, in MDAMB 231 cells, even though to a lesser extent than TBA, all four analogs inhibited the proliferation when tested at 10 and 30 µM at both 48 and 72 h, with a particularly increased effect at 30 µM after 72 h (inhibition percentage of about 20–30%) (Figure 4C,D).

### 2.5. Nucleolin Binding to G-Quadruplexes

**Figure 5:**
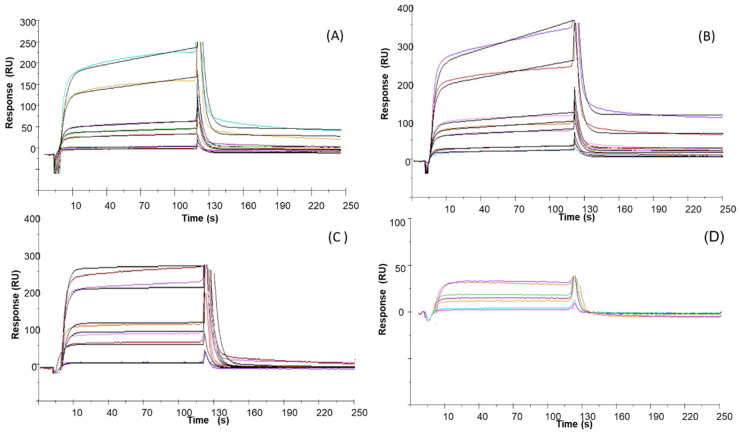
Overlay of experimental (colored lines, at different ligand concentrations) and the fitted (black lines) sensorgrams relative to SPR experiments for the binding to immobilized nucleolin of (**A**) TBAG4, (**B**) TBAG4GS, (**C**) TBAG3, and (**D**) T23.

The affinity of TBA and its analogs to NCL was investigated through SPR. NCL was efficiently immobilized on the CM5 chip, as already reported [24]. Dose-response profiles of the sensorgrams were reported in Figure 5 and Figure S5. Based on kinetic parameters reported in Table S1, dissociation constant (K_D_) values were evaluated and reported in Table 1. The comparison of the K_D_s indicated that TBA provided a high micromolar dissociation constant toward NCL while TBAG4, TBAG4GS, and TBAG41S analogs exhibited higher affinities and in particular, TBAG4 and TBAG4GS presented lower constants for dissociation (Figure 5A,B). Conversely, TBAG3 exhibited poor recognition ability toward NCL with a K_D_ value in the millimolar range (Figure 5C). T23 sequence, d(T)_23_, used as a negative control, provided a small dose-response signal variation (Figure 5D) and the failure of fitting of experimental sensorgrams.

## 3. Discussion

From a structural point of view, the introduction of extra G-tetrads in the “chair-like” G4 of TBA is able to affect the topology of the G-quadruplex adopted by the sequences. As a matter of fact, CD and PAGE results suggest that TBAG3 (containing a further G-tetrad than TBA) adopts a monomolecular parallel G-quadruplex structure. On the other hand, TBAG4 (containing two further G-tetrads than TBA) mainly adopts a monomolecular antiparallel G-quadruplex structure (Figure 1). Interestingly, the replacement of the loop residues with abasic sites in the loop regions of TBAG4 (namely, TBAG41S and TBAG4GS) affects both the molecularity and topology of the G4-structures. In fact, CD data indicate the presence of parallel G-quadruplexes for both sequences, while PAGE results suggest a coexistence of monomolecular and higher-order structures for TBAG4GS and the almost exclusive presence of higher-order G4-structures in the case of TBAG41S. CD heating experiments have highlighted the general enhancement of the stability for all G4s in comparison with TBA, as expected from the introduction of extra G-tetrads. However, it should be highlighted that the remarkable structural stability of TBAG4 shows a melting temperature higher than 90 °C.

As it is well known, TBA possesses anticoagulant properties being able to bind thrombin. However, none of the novel TBA analogs has shown anticoagulant activity. In a recent investigation, some authors have suggested that the recognition interface TBA/thrombin requires an antiparallel topology of the G4-structure core [21]. Therefore, since TBAG3, TBAG41S, and TBAG4GS mainly adopt parallel G4-structures, the absence of anticoagulant properties was expected to some extent. On the other hand, although TBAG4 preserves an antiparallel “chair-like” G4-structure similar to TBA, probably its larger size prevents an appropriate interaction with thrombin, thus resulting in no anticoagulant activity. However, as clarified before, the absence of anticoagulant properties should not be considered a disadvantage in view of the potential development of the investigated TBA derivatives as antiproliferative agents. The antiproliferative activity of the investigated ODNs was tested on both human prostate (DU 145) and breast (MDAMB 231) cancer cells. Although all TBA derivatives show an antiproliferative activity to the same extent, the most interesting data concern the results obtained from MDAMB 231 cells, which indicate a noteworthy activity for TBA, TBAG3, TBAG4, and TBAG4GS, revealing a reduction in cell viability percentage in the range 20–45%. Concerning TBAG3, it should be noted that it folds in a parallel G4-structure with three G-tetrads and three propeller loops, which is very similar to that adopted by other antiproliferative G-quadruplexes [25,26]. It is well known that TBA is highly susceptible to nucleases being degraded in a few hours [27], as also reported by us (Figure S3) [27]. Outcomes concerning the nuclease stability assay have indicated that all TBA derivatives significantly degrade in 72 h in serum, except TBAG4, which preserves its structure up to 72 h. It is interesting to note that there is a rough correlation between the degradability order (TBA < TBAG3 < TBAG41S ≤ TBAG4GS) and the antiproliferative activities in MDAMB 231 cells at 72 h (TBA < TBAG3 < TBAG4GS ≤ TBAG41S). The whole of the data strongly suggests that the observed antiproliferative activity of TBA, TBAG3, TBAG4GS, and TBAG41S could be mainly ascribed to the guanine-based degradation products, as reported by other authors on different G-rich ODNs [14,15]. On the contrary, the antiproliferative activity observed for TBAG4 could not depend on the guanine-based degradation products since this structure is characterized by an outstanding resistance to nuclease degradation. Furthermore, the absence of anticoagulant activity for TBAG4 excludes the interaction with thrombin and then the involvement of this protein in the antiproliferative activity. In order to verify if the observed antiproliferative activities could depend on the G4-NCL interaction to some extent, the affinities of all TBA derivatives to NCL were evaluated by SPR. Results indicate a significant affinity between TBAG4 and nucleolin. Taken together, our data suggest a potential involvement of NCL in the antiproliferative activity of TBAG4, although the involvement of other targets cannot be ruled out [28]. In a recent paper, some authors suggested that NCL preferentially binds long-looped G4-structures [29]. However, our investigations indicate that the repertoire of G4-structures able to significantly bind NCL should also include G4-structures with short loops, as in the case of TBAG4. The finding that TBAG4 possesses a promising antiproliferative activity against breast cancer cells and is able to bind efficiently with NCL is an unprecedented result that represents a significant advancement for the design of TBAG4 analogs endowed with NCL-mediated antiproliferative [18] and antiviral properties [24,30].

## 4. Materials and Methods

### 4.1. Oligonucleotide Synthesis and Purification

Modified ODNs reported in Table 1 were synthesized by an ABI 394 DNA synthesizer using solid phase β-cyanoethyl phosphoramidite chemistry at a 10 µmol scale. The syntheses were performed by using normal 3′-phosphoramidites and a 5′-dimethoxytrityl-3′-phosphoramidite-1′,2′-dideoxyribose (dSpacer, dS, Link Technologies, Glasgow, UK) for the introduction of abasic site mimic moieties in each sequence. The oligomers were detached from the support and deprotected by treatment with concentrated aqueous ammonia at 55 °C overnight. The combined filtrates and washings were concentrated under reduced pressure, redissolved in H_2_O, analyzed, and purified by high-performance liquid chromatography on a Nucleogel SAX column (Macherey-Nagel, 1000–8/46), using buffer A: 20 mM NaH_2_PO_4_/Na_2_HPO_4_ aqueous solution (pH 7.0), 20% (*v*/*v*) CH_3_CN and buffer B: 1 M NaCl, 20 mM NaH_2_PO_4_/Na_2_HPO_4_ aqueous solution (pH 7.0), 20% (*v*/*v*) CH_3_CN; a linear gradient from 0% to 100% B for 60 min and flow rate 1 mL/min were used. The fractions of the oligomers were collected and successively desalted by Sep-pak cartridges (C-18). The isolated oligomers proved to be >98% pure by NMR (Figures S6 and S7).

### 4.2. CD Spectroscopy

CD samples of modified oligonucleotides and their natural counterpart were prepared at an ODN concentration of 50 µM by using a potassium phosphate buffer (10 mM KH_2_PO_4_/K_2_HPO_4_, 70 mM KCl, pH 7.0) and submitted to the annealing procedure (heating at 90 °C and slowly cooling at room temperature). CD spectra of all quadruplexes and CD melting curves were registered on a Jasco 715 CD spectrophotometer. For the CD spectra, the wavelength was varied from 220 to 320 nm at 100 nm min^−1^ scan rate, and the spectra were recorded with a response of 16 s, at 2.0 nm bandwidth, and normalized by subtraction of the background scan with buffer. The temperature was kept constant at 20 °C with a thermoelectrically controlled cell holder (Jasco PTC-348). CD melting curves were registered as a function of temperature (range: 20–95 °C) for all quadruplexes at their maximum cotton effect wavelengths. The CD data were recorded in a 0.1 cm pathlength cuvette with a scan rate of 30 °C/h. Each measurement was the average of three scans.

### 4.3. Gel Electrophoresis

All oligonucleotides were analyzed by non-denaturing PAGE. Samples in the CD buffer (10 mM KH_2_PO_4_/K_2_HPO_4_, 70 mM KCl, pH 7.0) were loaded on a 20% polyacrylamide gel containing Tris–Borate-EDTA (TBE) 2.5× and KCl 20 mM. The run buffer was TBE 1× containing 50 mM KCl. For all samples, a solution of glycerol/TBE 10× was added just before loading. Electrophoresis was performed at 8 V/cm at a temperature close to 10 °C. Bands were visualized by UV shadowing.

### 4.4. Nuclease Stability Assay

Nuclease stability assay of modified ODNs was conducted in 10% Fetal Bovine Serum (FBS) diluted with Dulbecco’s Modified Eagle’s Medium (DMEM) at 37 °C and studied by CD analysis. Approximately 7 nmol of stock solution of each ODN (~1 O.D.U.) was evaporated to dryness under reduced pressure and then incubated with 250 μL 10% FBS at 37 °C. The degradation patterns were analyzed by monitoring the CD signal decrease in each sample at 37 °C as a function of time. CD spectra at 0, 24, 48, and 72 h for all ODNs, except for TBA (0, 15, 30 min), were recorded at 37 °C using a Jasco 715 spectrophotometer equipped with a Peltier temperature control system (Jasco, Tokyo, Japan). Data were collected from 240 to 320 nm with a 1 s response time and a 1 nm bandwidth using a 0.1 cm quartz cuvette. Each spectrum shown is corrected for the spectrum of the reaction medium (10% FBS in DMEM).

### 4.5. Prothrombin Time (PT) Assay

PT assay was performed on human plasma. The assay was measured by using Koagulab MJ Coagulation system with a specific kit, HemosIL RecombinPlasTin 2G (Instrumentation Laboratory, Milan, Italy). Briefly, this method relies on the high sensitivity of thromboplastin reagents based on recombinant human tissue factors. The addition of recombiplastin to the plasma, in the presence of calcium ions, initiates the activation of an extrinsic pathway that culminates with the conversion of fibrinogen into fibrin and, in turn, with a formation of a solid gel. In our experimental conditions, each ODN or vehicle was incubated with 100 µL of plasma at 37 °C for 15 min, and then 200 µL of the kit solution containing recombiplastin was added with the consequent activation of the extrinsic pathway. For the evaluation of PT at the concentration of 20 µM, 2 µL of the corresponding ODN solution (1 mM) or vehicle (phosphate buffer saline, PBS) was added to the suitable microtube. The PT was measured in triplicate, and the average and standard error values were calculated and expressed in seconds. The basal clotting time was evaluated by measuring the clotting time in the presence of a vehicle.

### 4.6. MTT Assay

Cell proliferation was measured by MTT (3-(4,5-dimethylthiazol-2-yl)-2,5-diphenyltetrazolium bromide) assay. Human prostate cancer cells (DU 145) and human breast cancer cells (MDAMB 231) were seeded on 96-well plates (3 × 10^3^ cells/well). After 24 h, cells were treated with TBA and its analogs for 48 and 72 h before adding MTT (cat. M5655, Merk, Italy) (final concentration 5 mg/mL in PBS). Cells were incubated for an additional 3 h at 37 °C. After this time, cells were lysed, and formazan salts resulting from MTT reduction were solubilized with a solution containing 50% (*v*/*v*) N,N-dimethyl formamide, 20% (*w*/*v*) sodium dodecylsulfate with an adjusted pH of 4.5. The absorbance was measured using a microplate spectrophotometer (Thermo Scientific Multiskan GO, Thermo Fisher Scientific, MA, USA) equipped with a 630-nm filter.

### 4.7. Surface Plasmon Resonance (SPR)

SPR assays were carried out using the SPR system BIAcore X-100 (GE Healthcare Milano, Italy). Human recombinant NCL (from OriGene Technologies, 0.18 µg/mL in 25 mM Tris.HCl, pH 7.3, 100 mM glycine, 10% glycerol) was immobilized on a CM5 sensor chip in acetate buffer (10 mM pH = 4, protein concentration 15 ng/mL) and a flow rate of 5 µL/min with an injection time of 7 min was implemented, as already reported [31,32]. The final immobilization level was 2346 RU. G-quadruplexes (stock solutions 1 mM) were diluted in running buffer (HEPES 10 mM and KCl 70 mM, pH 7.4), and binding experiments were performed at 25 °C with a flow equal to 30 µL/min, contact time equal to 2 min. The concentration range used was 5–500 µM. Reference channel signals were subtracted as blanks by employing the BIAevaluation program and the supplied two-state reaction equation. This model describes a 1:1 binding of the analyte to the immobilized ligand followed by a conformational change that stabilizes the complex.

## 5. Conclusions

In summary, we have investigated two TBA analogs containing one and two extra G-tetrads, namely TBAG3 and TBAG4, respectively, and two other derivatives in which one of the small loops at the bottom (TBAG41S) and the large loop at the top (TBAG4GS) of TBAG4 were completely modified by replacing all loop residues with abasic site mimics. Their antiproliferative activities against DU 145 and MDAMB 231 cancer cell lines, anticoagulant properties, biological degradability, and affinity to NCL were analyzed in comparison with TBA. CD and PAGE results suggest that TBAG3 adopts a monomolecular parallel G-quadruplex conformation, while TBAG4 mainly folds into a very stable monomolecular antiparallel structure. Interestingly, the presence of abasic site mimics in TBAG41S and TBAG4GS affects both molecularity and the topology of G4-structures. However, none of the investigated TBA derivatives exhibits anticoagulant activity, while all show similar antiproliferative activities. Results concerning the nuclease stability indicated that all TBA derivatives significantly degrade in 72 h in fetal bovine serum, except TBAG4, which preserves its structure up to 72 h, thus suggesting that its antiproliferative activity does not depend on the guanine degradation, differently from what observed for the other analogs. Although the involvement of other protein targets cannot be ruled out, the direct interaction between TBA and NCL, assessed through SPR assay, suggests a potential involvement of NCL in the antiproliferative activity of TBAG4, expanding the range of G4-structures able to significantly bind NCL and the possibility of developing novel anticancer and antiviral drugs.

## Figures and Tables

**Table 1:** Names, sequences, melting temperatures (T_m_), dissociation constant (K_D_) values for the interaction with NCL of TBA and its analogs. **S** indicates an abasic site mimic. See the experimental section for details.

NAME	SEQUENCE	T_m_ (°C) ± 1	K_D_ (µM)
**TBA**	GGTTGGTGTGGTTGG	50	62.5
**TBAG3**	GGGTTGGGTGTGGGTTGGG	77	1293
**TBAG4**	GGGGTTGGGGTGTGGGGTTGGGG	>90	13.5
**TBAG41S**	GGGG**SS**GGGGTGTGGGGTTGGGG	>70	41.5
**TBAG4GS**	GGGGTTGGGG**SSS**GGGGTTGGGG	85	32.2

## Data Availability

Not applicable.

## Supplementary Materials

The following supporting information can be downloaded at: https://www.mdpi.com/article/10.3390/ijms232314921/s1.
