# Supplementary material for: Properties and Potential Antiproliferative Activity of Thrombin-Binding Aptamer (TBA) Derivatives with One or Two Additional G-Tetrads
Source: Int J Mol Sci. 2022 Nov 29;23(23):14921. doi: 10.3390/ijms232314921 (PMC9736779; doi:10.3390/ijms232314921)
Supplement: Supplementary file 1 [file ijms-23-14921-s001.zip › ijms-2009784-supplementary.pdf]

SUPPLEMENTARY MATERIALS

Properties and Potential Antiproliferative Activity of Thrombin-Binding Aptamer (TBA) Derivatives with One or Two Additional G-Tetrads

Daniela Benigno †, Antonella Virgilio †, Ivana Bello, Sara La Manna, Valentina Vellecco, Mariarosaria Bucci, Daniela Marasco, Elisabetta Panza, Veronica Esposito * and Aldo Galeone *

Department of Pharmacy, University of Naples Federico II, I-80131 Napoli, Italy; daniela.benigno@unina.it (D.B.); antonella.virgilio@unina.it (A.V.); ivana.bello@unina.it (I.B.); sara.lamanna@unina.it (S.L.M.); vellecco@unina.it (V.V.); mrbucci@unina.it (M.B.); daniela.marasco@unina.it (D.M.); e.panza@unina.it (E.P.)

* Correspondence: verespos@unina.it (V.E.); galeone@unina.it (A.G.)

† These authors contributed equally to this work.

Table of contents

- CD melting profiles.
- PAGE analysis.
- CD spectra of TBA in 10% FBS.
- PT assay.
- Sensorgrams relative to SPR experiments.
- NMR spectra
- SPR kinetic parameters

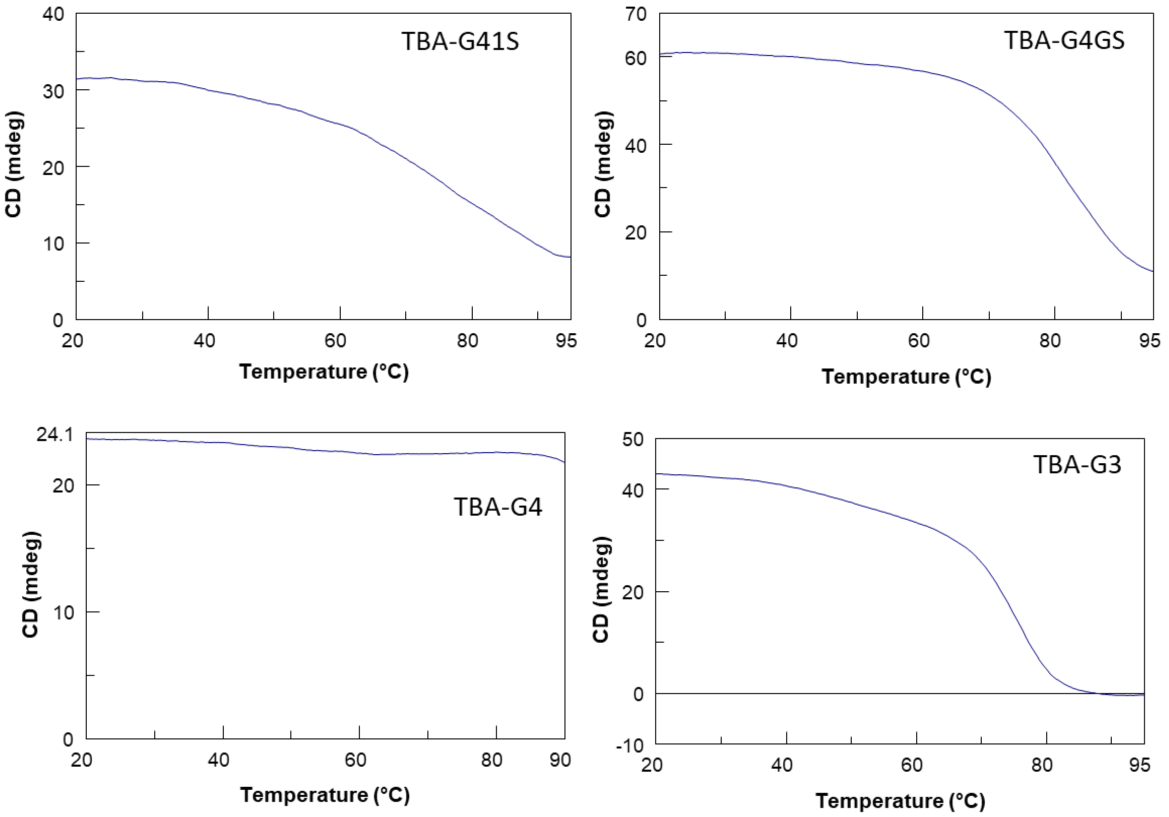

Figure S1. CD melting profiles of analyzed ODNs registered as a function of temperature for all modified G-quadruplexes at their maximum Cotton effect wavelengths. CD data were recorded in a 0.1 cm pathlength cuvette with a scan rate of 30°C/h at 50 μM ODN strand concentration in potassium phosphate buffer (10 mM KH_2PO_4/K_2HPO_4, 70 mM KCl, pH 7.0).

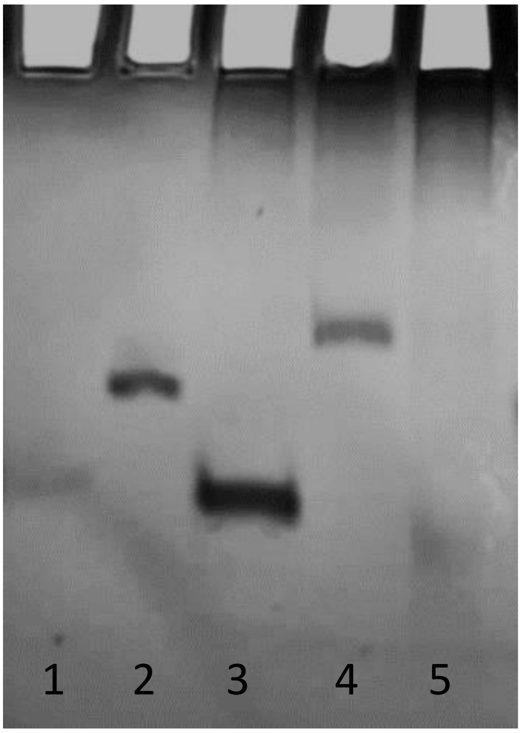

Figure S2. PAGE analysis of TBA and its investigated derivatives. Lane 1: TBA; lane 2: TBAG3; lane 3: TBAG4; lane 4: TBAG4GS; lane 5: TBAG41S. See Materials and Methods section for experimental details.

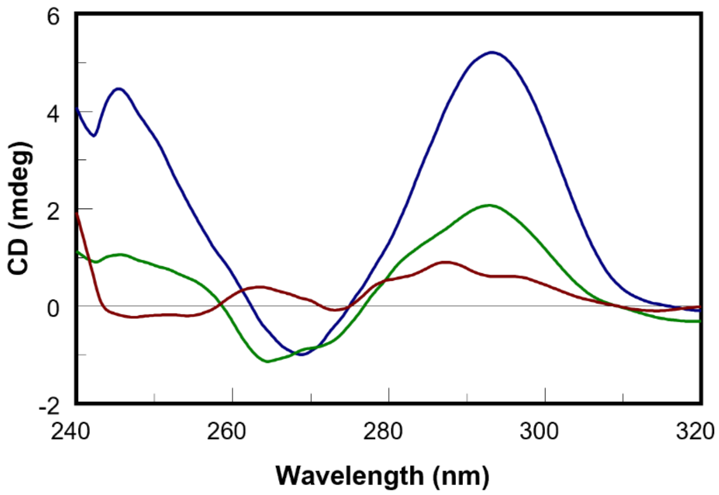

Figure S3. CD spectra of TBA in 10% FBS diluted with Dulbecco's Modified Eagle's Medium (DMEM), registered at 0 (blue), 15 min (green) and 30 min (red), at 37 °C.

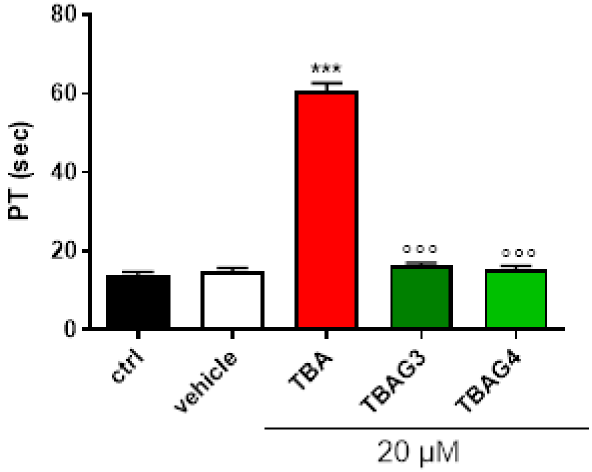

Figure S4. Effect of TBA and its analogues on PT assay. PT values were evaluated at 20 μM of ODN concentration and incubation time of 15 min. See Materials and Methods section for experimental details. Each experiment was run in triplicate. ***$p < 0.001$ vs. vehicle, °°° $p<0.001$ vs. TBA. The basal PT time is 14.7 ± 2.0 seconds.

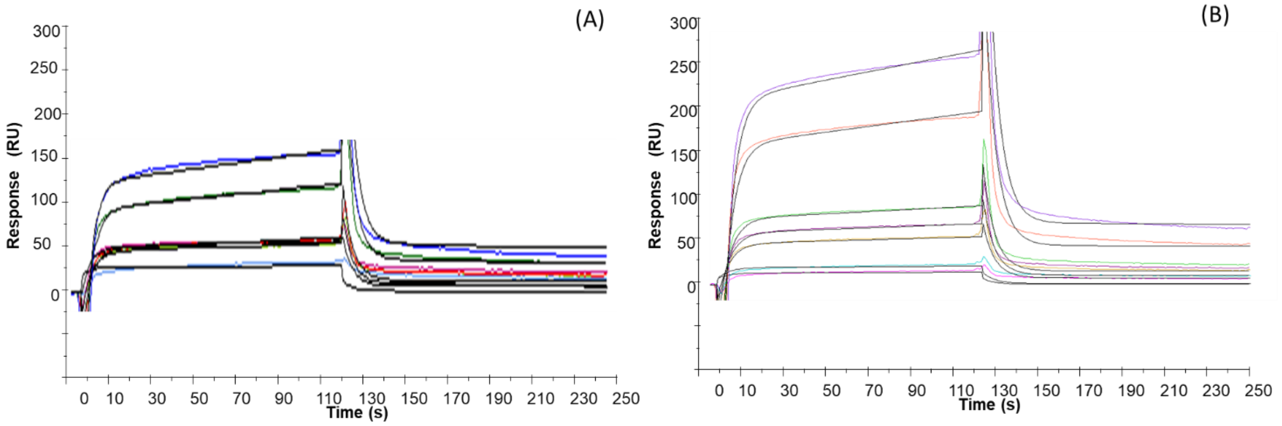

Figure S5. Overlay of experimental (colored lines, at different ligand concentrations) and the fitted (black lines) sensorgrams relative to SPR experiments for the binding to immobilized nucleolin of (A) TBA, (B) TBAG41S.

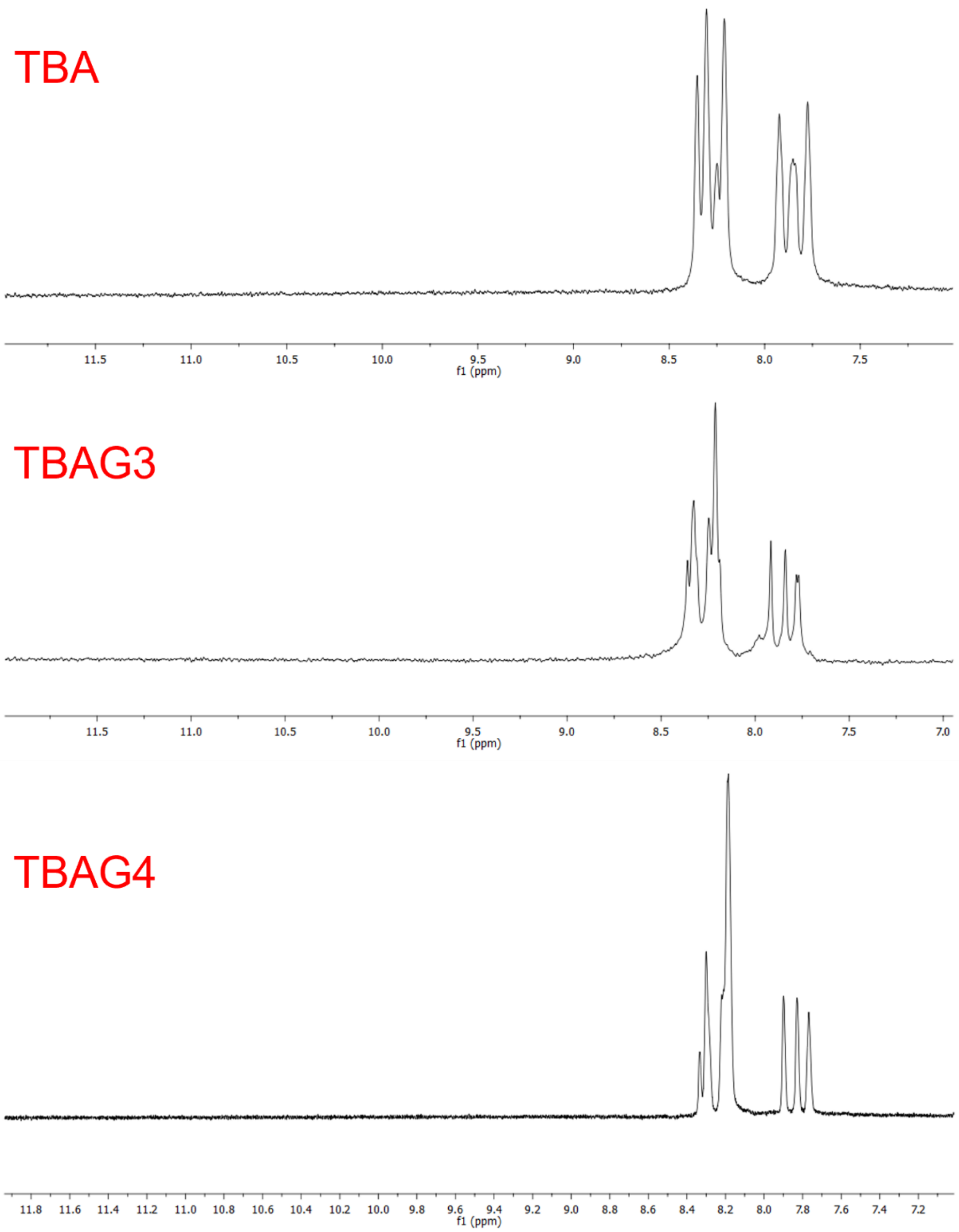

Figure S6. Imino and aromatic regions of the high resolution NMR spectra (700 MHz, D_2O, 75°C, no salt) of TBA, TBAG3 and TBAG4.

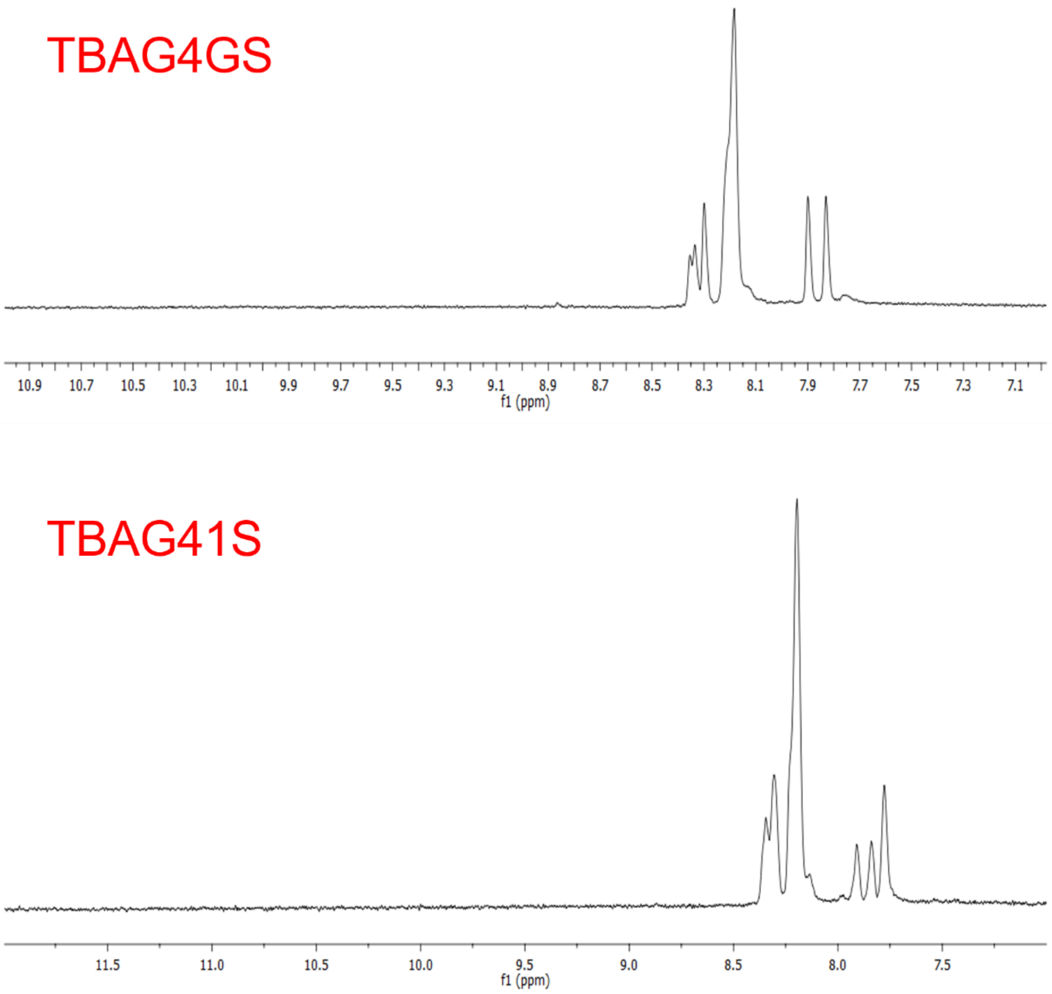

Figure S7. Imino and aromatic regions of the high resolution NMR spectra (700 MHz, D_2O, 75°C, no salt) of TBAG4GS and TBAG41S.

Table S1: SPR-based kinetic parameters for the interaction of NCL and TBA analogues using the X-100 software through Two State Reaction-Equation (k_{a1}: association rate constant for analyte binding, k_{d1} dissociation rate constant for analyte from the complex, k_{a2} forward rate constant and k_{d2} reverse rate constant for the conformational change).

Name	k_{a1} (1/Ms)	k_{d1} (1/s)	k_{a2} (1/Ms)	k_{d2}(1/s) *10^{-4}
**TBA**	169	0.232	9.72	6.25
**TBAG4**	967	0.248	9.20	8.40
**TBAG4GS**	391	0.274	14.1	0.710
**TBAG41S**	70.9	0.193	10.3	1.86
**TBAG3**	586	0.736	1.02	153
**T23**	No binding			
